# Age-Related Whole-Brain Structural Changes in Relation to Cardiovascular Risks Across the Adult Age Spectrum

**DOI:** 10.3389/fnagi.2019.00085

**Published:** 2019-04-24

**Authors:** Tao Gu, Chunyi Fu, Zhengyin Shen, Hui Guo, Meicun Zou, Min Chen, Kenneth Rockwood, Xiaowei Song

**Affiliations:** ^1^Department of Radiology, Beijing Hospital, National Center of Gerontology, Beijing, China; ^2^Health Research and Innovation, Surrey Memorial Hospital, Fraser Health Authority, Surrey, BC, Canada; ^3^SFU ImageTech Lab, Surrey Memorial Hospital, Surrey, BC, Canada; ^4^Department of Emergency Medicine, Beijing Hospital, National Center of Gerontology, Beijing, China; ^5^Department of Diagnostic Imaging, Tianjin Medical University General Hospital, Tianjin, China; ^6^Department of Medicine (Geriatric Medicine & Neurology), Dalhousie University, Halifax, NS, Canada; ^7^Centre for Healthcare of the Elderly, QEII Sciences Centre, Halifax, NS, Canada

**Keywords:** aging, Brain Atrophy and Lesion Index (BALI), cardiovascular risks, deficit accumulation, younger and older adults, magnetic resonance imaging (MRI)

## Abstract

**Background**: The brain atrophy and lesion index (BALI) has been developed to assess whole-brain structural deficits that are commonly seen on magnetic resonance imaging (MRI) in aging. It is unclear whether such changes can be detected at younger ages and how they might relate to other exposures. Here, we investigate how BALI scores, and the subcategories that make the total score, compare across adulthood and whether they are related to the level of cardiovascular risks, in both young and old adulthood.

**Methods**: Data were from 229 subjects (72% men; 24–80 years of age) whose annual health evaluation included a routine anatomical MRI examination. A BALI score was generated for each subject from T2-weighted MRI. Differences in the BALI total score and categorical subscores were examined by age and by the level of cardiovascular risk factors (CVRFs). Regression analysis was used to evaluate relationships between continuous variables. Relative risk ratios (RRRs) of CVRF on BALI were examined using a multinomial logistic regression. The area under the receiver operating characteristic (ROC) curve was used to estimate the classification accuracy.

**Results**: Nearly 90% of the participants had at least one CVRF. Mean CVRF scores increased with age (slope = 0.03; *r* = 0.36, 95% confidence intervals: 0.23–0.48; *p* < 0.001). The BALI total score was closely related to age (slope = 0.18; *r* = 0.69, 95% confidence intervals: 0.59–0.78; *p* < 0.001), as so were the categorical subscores (*r*’s = 0.41–0.61, *p* < 0.001); each differed by the number of CVRF (*t*-test: 4.16–14.83, *χ*^2^: 6.9–43.9, *p*’s < 0.050). Multivariate analyses adjusted for age and sex suggested an independent impact of age and the CVRF on the BALI score (for each year of advanced age, RRR = 1.20, 95% CI = 1.11–1.29; for each additional CVRF, RRR = 3.63, 95% CI = 2.12–6.23). The CVRF and BALI association remained significant even in younger adults.

**Conclusion**: The accumulation of MRI-detectable structural brain deficits can be evident from young adulthood. Age and the number of CVFR are independently associated with BALI score. Further research is needed to understand the extent to which other age-related health deficits can increase the risk of abnormalities in brain structure and function, and how these, with BALI scores, relate to cognition.

## Introduction

Magnetic resonance imaging (MRI) so often reveals age-related structural brain changes that “normal for age” is a common summary in imaging reports. Even so, some changes (e.g., atrophy and lacunes) are known to increase the risk of clinical consequences, whereas others, typically of a smaller scale (e.g., microbleeds, microinfarcts, and trace of white matter hyperintensities) receive less attention (Vernooij et al., 2007; Bos et al., 2016). With the recognition that, in aging, many small effects can add up to have major impacts on a range of adverse outcomes (Rockwood and Mitnitski, 2007), including cognition (Anstey and Dixon, 2014; Song et al., 2014; Canevelli et al., 2017), a concerted effort now suggests that such MRI detectable changes can have additive effects on brain structural and functional health (Pantoni et al., 2007; Gouw et al., 2008; Park et al., 2013; Tosto et al., 2014; Cai et al., 2015; Smith and Beaudin, 2018).

The Brain Atrophy and Lesion Index (BALI) has been developed to summarize several common structural changes in the brain (Zhang et al., 2011; Guo et al., 2014a,b; Grajauskas et al., 2018). These include gray matter lesions and subcortical dilated perivascular spaces, periventricular and white matter lesions, lesions in the basal ganglia and surrounding areas, lesions in the infratentorial compartment, and global atrophy (GA). The BALI has been validated in multiple independent datasets on subjects aged 55+ years, who were either with cognitively normal aging or mild cognitive decline and dementia. By late adulthood, BALI scores <0.2 (i.e., <20% of the BALI items being present), though rare, are associated with the best age-adjusted cognitive function (Zhang et al., 2011; Song et al., 2013; Guo et al., 2017).

Structural brain deficits, including atrophy, white matter changes and focal ischemic injury, have been linked both to a history of cerebrovascular disease and to cerebrovascular biomarkers (Pantoni et al., 2007; Bjerke et al., 2014; Poggesi et al., 2016; Grajauskas et al., 2019). Given both that these brain structural deficits accumulate with age (Good et al., 2001; Raz et al., 2010; Guo et al., 2014a,b), which continues to add explanatory power even in the face of cardiovascular risks (Debette et al., 2011; de Frias et al., 2014; McEvoy et al., 2015), and that across the life course, subclinical deficit appear to precede clinically detectable ones (Hickman et al., 2016; Blodgett et al., 2017), we hypothesized that age-related brain MRI changes are likely to be detectable at younger ages. Despite extensive evaluation of different age-related brain structural changes with hypertension and other cardiovascular risks (The LADIS Study Group et al., 2011; Rostrup et al., 2012; Bjerke et al., 2014), as far as we can determine, this proposition has not been evaluated using a summary quantitative score.

Here, our objectives were to investigate: (1) whole-brain MRI changes in adults over a wide age range; (2) how such changes are related to individual cardiovascular risks and the risk factors in combination in both younger and older adults.

## Materials and Methods

### Participants

We accessed data from a consecutive series of anonymous company employees (current and retired) who underwent a general health evaluation at Beijing Hospital from August 16, 2016 to August 31, 2017 and who agreed to have a cranial MRI scan (*n* = 248). Each subject included in the study had a clinical interview, physical examination, blood sample test, and a standard anatomical MRI of the brain. Subjects who were diagnosed as having terminal malignancy, stroke, heart diseases, or cognitive decline (*n* = 19) were excluded from the study, yielding a sample size *n* = 229 participants for further analysis.

### Processing of the Cardiovascular Risk Factors

Five commonly identified cardiovascular risk factors (CVRFs) including hypertension, diabetes, dyslipidemia, body-mass abnormality, and smoking (Khot et al., 2003; Yusuf et al., 2014), were retrieved from the clinical interview and the blood test data. Each item was processed being normal (code as “0”) vs. abnormal (coded as “1”) based on standard criteria as described below: (1) arterial hypertension: systolic blood pressure >130 mmHg, diastolic blood pressure <80 mm Hg, or use of antihypertension medication; (2) diabetes mellitus: fasting serum glucose level >7.0 mmol/L or treatment with insulin or oral hypoglycemic medication; (3) dyslipidemia: total cholesterol level >5.2 mmol/L, triglyceride level >1.7 mmol/L, or use of lipid-lowering drugs; (4) body mass index (BMI) >25; and (5) being a current smoker. Information regarding basic demographics of the subjects including age, sex, education level, retirement status, was also obtained.

### MRI Tests

MRI scans were acquired using one of four MRI scanners, including two of 3.0-Tesla (Discovery MR750, General Electric Medical Systems, Chicago, IL, USA and Acheiva, Philips Medical Systems, Best, Netherlands) and two of 1.5-Tesla (MAGNETOM Espree, Siemens, Germany and Optima MR360, General Electric Medical Systems, Chicago, IL, USA). Nearly three quarters (73.8%) of the MRI data were acquired with 3.0T. 2D T2-weighted images (T2WI) were used for evaluation, which were acquired with the same resolution as with the T1-weighted images in this dataset. Parameter settings were: TR/TE = 2500–5600/90–110 ms, flip angle = 90° or 140–160°, field of view = 230 × 230 mm, matrix = 180 × 256, slice thickness = 5.0 mm no gap, 24 axial slices to cover the whole brain.

### Evaluation of the Brain Atrophy and Lesion Index

As described elsewhere, the BALI is a semi-quantitative summary rating scale (Zhang et al., 2011; Guo et al., 2014a,b), adapted from several well-established scales that assess localized structural changes commonly seen in the aging brain (Fazekas et al., 1987; Scheltens et al., 1993). Changes in the following categories are integrated in the BALI evaluation: (1) gray matter lesions (e.g., cortical infarcts) and subcortical dilated perivascular spaces (GM-SV), (2) deep white matter lesions (DWM), (3) periventricular white matter lesions (PV), (4) lesions in the basal ganglia and surrounding areas (BG), (5) lesions in the infratentorial compartment (IT), (6) Global Atrophy, and (7) other findings (Guo et al., 2014a,b). Applying the BALI rating schema, a value between 0 and 3 was assigned to assess a change in each category, with a higher score meaning greater severity. In the categories (DWM and GA), values of 4–5 were also used, allowing capture of more severe changes and thereby avoiding ceiling effects. The “other findings” category was included to record the possible changes such as neoplasm, trauma, idiopathic normal-pressure hydrocephalus, focal asymmetry, and deformity, each of which is sometimes seen in older adults (Guo et al., 2017).

The BALI total score was calculated as the sum of subscores of all the seven categories, and so ranges from 0/25 (no structural deficits) to a theoretical 25/25 (or 1.0), although in practice BALI scores >18/25 (or 0.7) are rare. Two experienced neuroradiologists (TG, HG) performed the evaluation independently, with the subjects’ demographic information masked. The first author, TG evaluated and scored all the images, and HG assessed a randomly selected 20% images to test interrater reliability of the rating, as reported elsewhere, together with estimates of intra-rater reliability (Gu et al., 2018).

### Statistical Analysis

Analysis of Variance (ANOVA) and non-parametric Kruskal-Wallis test were used to test the mean difference in BALI total and categorical subscores between subjects of different age groups and different numbers of CVRF. Student *t*-test was used to compare the scores between two groups of subjects with vs. without a given CVRF. Differences in the demographics variable among subjects with different BALI categorical subscores were examined using either ANOVA (for interval variables) or *χ*^2^ test (for categorical variables). The relationship between age and the BALI total score was examined using regression models; i.e., linear and exponential as with previous publications (Song et al., 2013; Guo et al., 2014b). Spearman correlation was used to examine the associations of the BALI scores with the number of CVRF. A multivariable multinomial logistic regression model was used to examine the association of the increments of the CVRF exposures on the BALI scores, for which a 3-level categorical variable representing the tertiles was used as outcome; and age, sex, education, marriage, occupation, and MR scanner and field intensity (3T vs. 1.5 T) were adjusted. The relative risk ratio (RRR) was presented with the 95% confidence intervals (CI). The analysis was conducted for the entire sample and separately for the younger and older subgroups divided at age of 50 years, with the rationale that: (1) it is close to the median age of 47 in this sample, and (2) age-related brain changes on MRI typically become evident from the fifth decade (Good et al., 2001; Grajauskas et al., 2019). Similarly, the area under the curve (AUC) of receiver operating characteristic (ROC) analysis was conducted for the various groups using tertiled BALI scores, to evaluate the accuracy of the number of CVRF in identifying individuals in different tertiles.

Statistical analyses and result presentation were performed using IBM Statistics SPSS version 23, StataCorp Stata version 13, and Mathworks Matlab version 2013a. Statistical significance level was set at *p* < 0.050.

## Results

Participants (men = 165, women = 64) were aged between 24 and 80 years (mean age = 48.3 ± 12.5 years; median = 47 years); most were married (95%), had received a college or university degree (91%), and were employed when evaluated (83%). Only 11% subjects were free of any of the risk factors; 29%, 18%, 11%, and 1%, respectively had 2–5 of the CVRF under consideration. Mean CVRF scores increased with age (slope = 0.03; *r* = 0.36; 95% confidence intervals: 0.23–0.48). Subjects who had all five CVRF were significantly older than those who had none or just one of them, whereas those with 2–4 CVRF did not differ in their mean age. More subjects had hyperlipidemia (60%) or abnormal BMI (57%), whereas 34% and 7% had hypertension and diabetes mellitus, and 27% were current smokers (Tables s 1a–c).

**Table 1a:** Demographics by categorical scores of the Brain Atrophy and Lesion Index for All (*n* = 229).

BALI category	Scores	Subject (N)	Age (year)	Male (%)	Hypertension (%)	Diabetic Mellitus (%)	Hyper-lipidemia (%)	BMI >25 (%)	Current Smoker (%)
GM-SV	0	5	32.8 ± 3.6 (28–36)	40.0	0.0	0.0	20.0	40.0	40.0
	1	124	44.8 ± 10.8 (24–78)	74.2	18.5	4.0	58.0	50.8	26.6
	2	97	52.8 ± 12.3 (26–80)	73.2	53.6	10.3	64.9	64.9	27.8
	3	3	72.7 ± 7.8 (64–79)	66.7	100.0	33.3	66.7	66.7	0.0
	*F/x*^2^ *(p)*		16.77* (0.000)	2.67* (0.445)	38.18* (0.00)	6.89 (0.075)	4.58 (0.206)	5.13 (0.162)	1.58 (0.664)
DMW	0	42	40.1 ± 9.2 (25–60)	64.3	19.0	0.0	52.3	45.2	26.2
	1	62	45.8 ± 9.9 (24–74)	67.7	20.9	0.0	54.8	54.8	25.8
	2	118	51.5 ± 12.8 (26–80)	77.1	43.2	11.0	65.3	60.2	28.0
	3	7	65.4 ± 11.2 (44–79)	71.4	85.7	42.9	71.4	85.7	28.6
	*F/x*^2^ *(p)*		16.60* (0.000)	3.34 (0.343)	21.67* (0.000)	24.62* (0.000)	3.44 (0.328)	5.32 (0.150)	0.12 (0.989)
PV	0	139	43.6 ± 9.9 (24–71)	71.9	21.6	5.0	56.1	54.0	28.8
	1	70	52.4 ± 11.8 (26–79)	77.1	44.3	5.7	67.1	57.1	25.7
	2	18	65.9 ± 10.0 (45–80)	55.6	83.3	27.8	61.1	72.2	22.2
	3	2	71.5 ± 7.8 (66–77)	50.0	100.0	0.0	100	100.0	0.0
	*F/x*^2^ *(p)*		32.65* (0.000)	3.82 (0.282)	36.22* (0.000)	13.11* (0.004)	3.71 (0.295)	3.73 (0.293)	1.23 (0.747)
BG	0	60	42.9 ± 10.6 (24–64)	66.7	13.3	1.6	50.0	50.0	23.3
	1	62	44.2 ± 10.2 (28–76)	69.4	17.7	1.6	59.7	48.4	27.4
	2	96	52.8 ± 12.4 (29–80)	77.1	52.1	12.5	66.7	63.5	26.0
	3	11	61.6 ± 11.9 (40–79)	72.7	81.8	18.2	63.6	81.8	54.5
	*F/x*^2^ *(p)*		17.20* (0.000)	2.30 (0.513)	43.88* (0.000)	11.98* (0.007)	4.34 (0.227)	7.50 (0.058)	4.69 (0.196)
IT	0	85	42.9 ± 10.6 (24–72)	69.4	14.1	1.2	49.4	45.9	28.2
	1	68	46.2 ± 11.6 (29–78)	66.2	35.3	8.8	61.8	55.9	20.6
	2	66	56.3 ± 11.4 (32–80)	81.8	56.1	12.1	68.2	68.2	31.8
	3	10	55.6 ± 12.1 (36–76)	70.0	50.0	10.0	90.0	80.0	30.0
	*F/x*^2^ *(p)*		20.03* (0.000)	4.61 (0.203)	30.45* (0.000)	7.59 (0.055)	9.67* (0.022)	9.83* (0.020)	2.30 (0.512)
GA	0	72	41.2 ± 9.3 (24–72)	61.1	13.9	1.4	48.6	47.2	22.2
	1	103	46.4 ± 9.4 (25–71)	74.8	29.1	3.9	63.1	54.4	29.1
	2	48	60.1 ± 11.0 (32–78)	83.3	68.7	18.7	75.0	70.8	31.3
	3	4	69.3 ± 10.5 (54–77)	75.0	75.0	50.0	50.0	100.0	25.0
	4	2	79.5 ± 0.7 (79–80)	50.0	100.0	0.0	0.0	100.0	0.0
	*F/x*^2^ *(p)*		37.86* (0.000)	8.19 (0.085)	46.74* (0.000)	26.76* (0.000)	11.99* (0.017)	11.35* (0.023)	2.25 (0.689)
Others	0	219	48.2 ± 12.5 (24–80)	71.2	33.3	7.3	58.9	57.1	27.4
	1	10	50.9 ± 12.7 (32–64)	90.0	50.0	0.0	90.0	50.0	20.0
	*F/x*^2^ *(p)*		0.45 (0.503)	1.67 (0.196)	1.18 (0.277)	0.79 (0.375)	3.86 (0.049)	0.20 (0.659)	0.27 (0.607)

**Table 1b:** Demographics by categorical scores of the Brain Atrophy and Lesion Index for Old Group (*n* = 97).

BALI category	Scores	Subject (N)	Age (year)	Male (%)	Hypertension (%)	Diabetic Mellitus (%)	Hyper-lipidemia (%)	BMI >25 (%)	Current Smoker (%)
GM-SV	0	0	-	0	0	0	0	0	0
	1	37	57.8 ± 6.4 (50–78)	67.6	27.0	5.4	67.6	51.4	27.0
	2	57	60.9 ± 8.6 (50–80)	71.9	66.7	17.5	64.9	73.7	24.6
	3	3	72.7 ± 7.8 (64–79)	66.7	100.0	33.3	66.7	66.7	0.0
	*F/x*^2^ * (p)*		16.77* (0.000)	0.22 (0.895)	16.93* (0.00)	3.91 (0.142)	0.07 (0.965)	4.92 (0.085)	1.09 (0.580)
DMW	0	7	54.4 ± 3.6 (50–60)	42.9	14.3	0.0	57.1	57.1	28.6
	1	23	55.7 ± 5.4 (50–74)	65.2	34.8	0.0	56.5	47.8	30.4
	2	61	61.5 ± 8.4 (50–80)	75.4	60.7	16.4	70.5	70.5	21.3
	3	6	69.0 ± 6.5 (64–79)	66.7	83.3	50.0	66.7	83.3	33.3
	*F/x*^2^ * (p)*		20.134* (0.000)	3.59 (0.309)	10.91* (0.012)	12.04* (0.007)	1.71 (0.634)	4.86 (0.182)	1.08 (0.782)
PV	0	40	55.8 ± 4.9 (50–71)	75.0	27.5	10.0	57.5	65.0	30.0
	1	39	60.6 ± 8.1 (50–79)	74.4	64.1	10.3	76.9	61.5	25.6
	2	16	68.3 ± 7.5 (50–80)	50.0	81.3	31.3	56.3	68.8	12.5
	3	2	71.5 ± 7.8 (66–77)	50.0	100.0	0.0	100.0	100.0	0.0
	*F/x*^2^ * (p)*		28.295* (0.000)	4.27 (0.234)	19.25* (0.000)	5.43 (0.143)	5.07 (0.167)	1.38 (0.710)	2.56 (0.465)
BG	0	19	55.1 ± 4.6 (50–64)	57.9	21.1	0.0	73.7	63.2	21.1
	1	22	55.6 ± 5.6 (50–76)	81.8	31.8	4.6	72.7	50.0	31.8
	2	47	63.1 ± 8.3 (50–80)	70.2	68.1	21.3	61.7	70.2	19.2
	3	9	65.6 ± 8.8 (54–79)	66.7	88.9	22.2	55.6	77.8	44.4
	*F/x*^2^ * (p)*		26.109* (0.000)	2.84 (0.417)	20.67* (0.000)	7.54 (0.056)	1.77 (0.622)	3.41 (0.333)	3.40 (0.334)
IT	0	22	56.5 ± 6.2 (50–72)	68.2	27.3	4.6	68.2	50.0	27.3
	1	22	59.7 ± 8.2 (50–78)	54.6	50.0	13.6	59.1	54.6	18.2
	2	47	61.5 ± 8.7 (51–80)	80.9	63.8	17.0	66.0	76.6	27.7
	3	6	63.7 ± 7.3 (54–76)	50.0	66.7	16.7	83.3	66.7	16.7
	*F/x*^2^ * (p)*		2.38 (0.074)	6.33 (0.097)	8.57* (0.036)	2.07 (0.557)	1.32 (0.725)	6.01 (0.111)	1.01 (0.799)
GA	0	16	54.1 ± 5.3 (50–72)	50.0	12.5	0.0	62.5	56.3	18.8
	1	36	56.6 ± 4.5 (51–71)	69.4	38.9	5.6	69.4	50.0	25.0
	2	39	63.9 ± 7.7 (50–78)	79.5	76.9	23.1	69.2	76.9	28.2
	3	4	69.3 ± 10.5 (54–77)	75.0	75.0	50.0	50.0	100.0	25.0
	4	2	79.5 ± 0.7 (79–80)	50.0	100.0	0.0	0.0	100.0	0.0
	*F/x*^2^ * (p)*		36.06* (0.000)	5.16 (0.271)	24.89* (0.000)	12.46* (0.014)	4.80 (0.309)	9.76* (0.045)	1.22 (0.875)
Others	0	92	60.0 ± 8.4 (50–80)	69.6	52.2	14.1	65.2	67.4	26.1
	1	5	61.8 ± 3.0 (58–64)	80.0	60.0	0.0	80.0	20.0	0.0
	*F/x*^2^ * (p)*		1.26 (0.262)	0.246 (0.620)	0.117 (0.733)	0.816 (0.366)	0.462 (0.497)	4.68* (0.031)	1.73 (0.188)

**Table 1c:** Demographics by categorical scores of the Brain Atrophy and Lesion Index for Young Group (*n* = 132).

BALI category	Scores	Subject (N)	Age (year)	Male (%)	Hypertension (%)	Diabetic Mellitus (%)	Hyper-lipidemia (%)	BMI >25 (%)	Current Smoker (%)
GM-SV	0	5	32.8 ± 3.6 (28–36)	40.0	100.0	0.0	20.0	40.0	40.0
	1	87	39.3 ± 6.9 (24–49)	74.7	85.1	3.5	54.0	55.2	26.4
	2	40	41.4 ± 6.0 (26–49)	75.0	65.0	0.0	65.0	62.5	32.5
	3	0	-	0.0	0.0	0.0	0.0	0.0	0.0
	*F/x*^2^ *(p)*		4.28* (0.016)	2.99 (0.224)	8.11* (0.017)	1.59 (0.452)	4.08 (0.130)	1.20 (0.549)	0.81 (0.667)
DMW	0	35	37.3 ± 7.0 (25–48)	68.6	20.0	0.0	51.4	48.6	25.7
	1	39	39.9 ± 6.7 (24–49)	69.2	12.8	0.0	53.9	64.1	23.1
	2	57	40.8 ± 6.2 (26–49)	79.0	24.6	5.3	59.7	56.1	35.1
	3	1	44.0 ± na (44–44)	100.0	100.0	0.0	100.0	100.0	0.0
	*F/x*^2^ *(p)*		2.26* (0.085)	2.03 (0.566)	5.88 (0.118)	4.04 (0.257)	1.46 (0.691)	2.58 (0.460)	2.29 (0.515)
PV	0	99	38.8 ± 6.7 (24–49)	70.7	19.2	3.0	55.6	54.6	28.3
	1	31	42.0 ± 6.2 (26–49)	80.7	19.4	0.0	54.8	61.3	25.8
	2	2	46.5 ± 2.1 (45–48)	100.0	100.0	0.0	100.0	100.0	100.0
	3	0	-	0.0	0.0	0.0	0.0	0.0	0.0
	*F/x*^2^ *(p)*		4.09* (0.019)	1.93 (0.381)	7.90* (0.019)	1.02(0.600)	1.60 (0.450)	1.98 (0.371)	5.09 (0.078)
BG	0	41	37.3 ± 7.2 (24–48)	70.7	9.8	2.4	39.0	53.7	24.4
	1	40	37.9 ± 5.6 (28–49)	62.5	10.0	0.0	52.5	52.5	25.0
	2	49	42.8 ± 5.8 (29–49)	83.7	36.7	4.1	71.4	61.2	32.7
	3	2	44.0 ± 5.7 (40–48)	100.0	50.0	0.0	100.0	100.0	100.0
	*F/x*^2^ *(p)*		7.58* (0.000)	6.00 (0.113)	14.6* (0.002)	1.70 (0.636)	11.3* (0.010)	2.38 (0.498)	5.97 (0.113)
IT	0	63	38.2 ± 7.2 (24–49)	69.8	9.5	0.0	42.9	50.8	28.6
	1	46	39.7 ± 6.0 (29–49)	71.7	28.3	6.5	63.0	60.9	21.7
	2	19	43.4 ± 5.4 (32–49)	84.2	36.8	0.0	73.7	57.9	42.1
	3	4	43.5 ± 5.2 (36–47)	100.0	25.0	0.0	100.0	100.0	50.0
	*F/x*^2^ *(p)*		3.68* (0.014)	3.07 (0.382)	89.54* (0.023)	5.74 (0.125)	10.9* (0.012)	4.29 (0.232)	3.64 (0.303)
GA	0	56	37.5 ± 6.5 (24–49)	64.3	14.3	1.8	44.6	48.2	23.2
	1	67	40.9 ± 6.4 (25–49)	77.6	23.9	3.0	59.7	61.2	31.3
	2	9	43.3 ± 6.5 (32–49)	100.0	33.3	0.0	100.0	77.8	44.4
	3	0	-	0.0	0.0	0.0	0.0	0.0	0.0
	4	0	-	0.0	0.0	0.0	0.0	0.0	0.0
	*F/x*^2^ *(p)*		6.09* (0.003)	6.27* (0.044)	2.71 (0.258)	0.42 (0.810)	10.38* (0.006)	3.82 (0.148)	2.14 (0.343)
Others	0	127	39.6 ± 6.7 (24–49)	72.4	19.7	2.4	54.3	55.9	28.4
	1	5	40.0 ± 7.5(32–48)	100.0	40.0	0.0	100.0	80.0	40.0
	*F/x*^2^ *(p)*		0.01 (0.904)	1.88 (0.171)	1.22 (0.269)	0.12 (0.728)	4.07* (0.044)	1.14 (0.286)	0.32 (0.572)

Note: data are presented as mean ± standard deviation unless specified otherwise.; GM-SV, gray matter and subcortical lesions—subcortical dilated perivascular spaces; DWM, deep white matter lesions; PV, periventricular white matter lesions; BG, lesions in the basal ganglia and surrounding areas; IT, lesions in the infratentorial regions; GA, global atrophy. BMI, body mass index; BALI, Brain Atrophy and Lesion Index; *p < 0.05.

**Figure 1:**
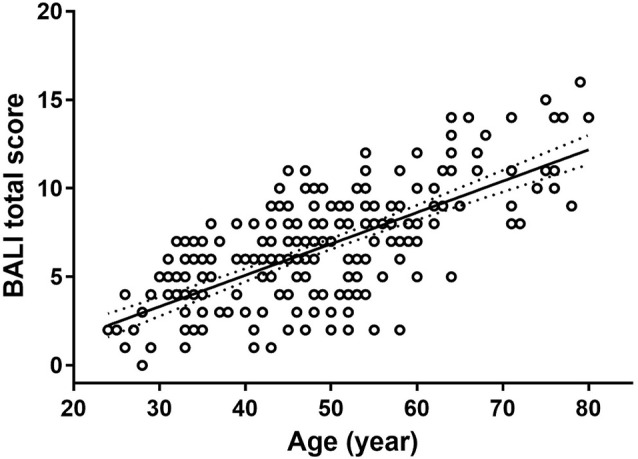
The Brain Atrophy and Lesion Index (BALI) as a function of age. Circles represent the raw data of the BALI total scores averaged by each year of age. Solid line represents the curve fitting using an exponential function; dotted lines represent the 95% confidence intervals. The relationship was described by a linear regression *y = a+bx*, where *x* is age, *y* is BALI score, *a* = −1.99 (−3.21 – −0.78);* b* = 0.18 (0.15–0.20);* r =* 0.69 (0.59–0.78), with 95% confidence interval given in the bracket. *p* < 0.001.

To execute Objective 1 (whole-brain MRI changes in adults over the age range), we found that the BALI total score was closely related to age (slope = 0.18; 95% CI: 0.15–0.20; *r* = 0.69, 95% CI = 0.59–0.78, *p* < 0.001), increased from an average of 0.08 (i.e., 2/25) at age 24 years to 0.48 (i.e., 12/25) at age 80 years (Figure 1). The relationship could be fitted equally well with an exponential function f(x) = a• exp(b•x), where *a* = 1.859, *b* = 0.025, *r* = 0.69, *p* < 0.001.

**Figure 2:**
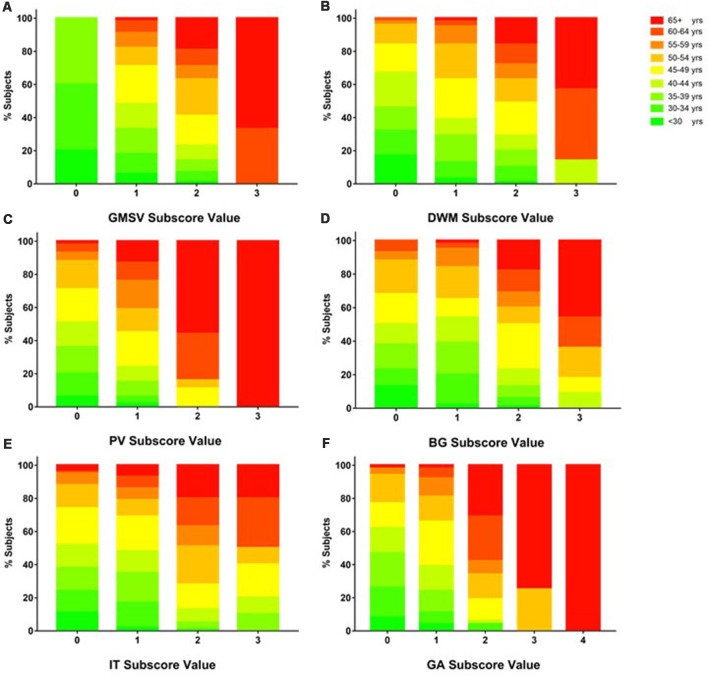
Proportion of subjects in each age group as grouped by BALI subscore values. Data for each subscore are presented using a sub-figure. **(A)** GM-SV, gray matter and subcortical lesions—subcortical dilated perivascular spaces. **(B)** DWM, deep white matter lesions. **(C)** PV, periventricular white matter lesions. **(D)** BG, lesions in the basal ganglia and surrounding areas. **(E)** IT, lesions in the infratentorial regions. **(F)** GA, global atrophy. In each sub-figure, stacked color bars present different age groups. Refer Supplementary Figure S2 for additional data and statistics of the age and BALI subscores relationships.

The mean subscore of each BALI category showed an increase with an age increment (Supplementary Figure S1). The GMSV change appeared the earliest (e.g., <30 years of age); this was followed by the change of the DWM, BG, and IT category, which started to accumulate at 30–35 years; in contrast, the GA and PV subscores became most significant only at older ages, e.g., 60+ years (Supplementary Figure S1). For each BALI subscore, there was a statistically significant trend demonstrating that subjects in older age groups were more likely to have a higher subscore value (*χ*^2^ ranged between 62.1 for IT and 144.50 for GA, *p* < 0.001; Figure 2; Supplementary Figures S2, S3).

To execute Objective 2 (brain changes in relation to CVRFs, individually and in combination, in both younger and older adults), we found that on average, subjects who had a higher subscore with any of the BALI categories (except for “Others”, seen in 4.4%) were older (*F*’s = 16.60–37.86, *p* < 0.001) and more likely to have hypertension (*χ*^2′^s: 21.67–43.88, *p* < 0.05), diabetes mellitus (*χ*^2′^s: 6.89–26.76, *p* < 0.05), and other CVRF (Table 1a). Similarly, subjects with any CVRF, especially hypertension, diabetes, and high BMI, often showed a higher mean BALI total score and subscore than those who did not have the CVRF (Table 2a). Virtually, this observation was also held true in both the younger and older subgroups when analyzed separately (Tables s 1b,c, 2b,c).

**Table 2a:** BALI categorical and total scores by Cardiovascular Risk Factors for All (*n* = 229).

BALI category		GMSV	DWM	PV	BG	IT	GA	Others	Total
Hypertension	No (n)	1.26 ± 0.51	1.23 ± 0.80	0.30 ± 0.50	0.99 ± 0.84	0.77 ± 0.87	0.70 ± 0.67	0.03 ± 0.18	5.29 ± 2.49
	Yes (n)	1.74 ± 0.52	1.71 ± 0.76	0.86 ± 0.82	1.77 ± 0.79	1.45 ± 0.83	1.45 ± 0.86	0.06 ± 0.25	9.04 ± 3.03
	*T(p)*	6.66* (0.000)	4.31* (0.000)	6.42* (0.000)	6.82* (0.000)	5.62* (0.000)	7.22* (0.000)	1.09 (0.279)	10.01* (0.000)
Diabetic Mellitus	No	1.40 ± 0.56	1.33 ± 0.81	0.46 ± 0.65	1.20 ± 0.90	0.96 ± 0.92	0.90 ± 0.79	0.05 ± 0.21	6.31 ± 3.10
	yes	1.75 ± 0.58	2.19 ± 0.40	0.88 ± 0.89	1.94 ± 0.68	1.56 ± 0.73	1.75 ± 0.78	0.00 ± 0.00	10.06 ± 2.77
	*T(p)*	2.40* (0.017)	4.17* (0.000)	2.38* (0.018)	3.21* (0.002)	2.56* (0.011)	4.15* (0.000)	−0.88 (0.378)	4.71* (0.000)
Hyperlipidemia	No	1.35 ± 0.58	1.27 ± 0.83	0.41 ± 0.63	1.11 ± 0.92	0.78 ± 0.84	0.84 ± 0.90	0.01 ± 0.11	5.77 ± 3.35
	Yes	1.48 ± 0.54	1.47 ± 0.80	0.54 ± 0.71	1.35 ± 0.88	1.15 ± 0.94	1.04 ± 0.76	0.07 ± 0.25	7.09 ± 3.02
	*T(p)*	1.67 (0.095)	1.79 (0.076)	1.50 (0.136)	1.97 (0.050)	3.06* (0.002)	1.82 (0.070)	1.97 (0.050)	3.11* (0.002)
BMI > 25	No	1.33 ± 0.55	1.26 ± 0.83	0.40 ± 0.59	1.09 ± 0.86	0.79 ± 0.85	0.76 ± 0.69	0.05 ± 0.22	5.69 ± 2.80
	yes	1.50 ± 0.56	1.49 ± 0.80	0.55 ± 0.74	1.38 ± 0.92	1.17 ± 0.93	1.11 ± 0.88	0.04 ± 0.19	7.24 ± 3.36
	*T(p)*	2.24* (0.026)	2.12* (0.035)	1.66 (0.098)	2.40* (0.017)	3.19* (0.002)	3.27* (0.001)	0.44 (0.660)	3.72* (0.000)
Current Smoker	No	1.44 ± 0.57	1.38 ± 0.82	0.51 ± 0.70	1.21 ± 0.88	0.99 ± 0.90	0.93 ± 0.84	0.05 ± 0.21	6.51 ± 3.26
	Yes	1.40 ± 0.56	1.42 ± 0.82	0.42 ± 0.62	1.37 ± 0.95	1.05 ± 0.97	1.02 ± 0.76	0.03 ± 0.18	6.71 ± 3.11
	*T(p)*	0.41 (0.686)	0.30 (0.767)	0.95 (0.345)	1.21 (0.229)	0.44 (0.658)	0.671 (0.503)	0.51 (0.609)	0.41 (0.685)
Number of CVRFs	0	1.16 ± 0.47	1.04 ± 0.90	0.12 ± 0.33	0.88 ± 0.78	0.56 ± 0.71	0.48 ± 0.51	0.00 ± 0.00	4.24 ± 2.13
	1	1.26 ± 0.53	1.17 ± 0.82	0.37 ± 0.54	0.96 ± 0.84	0.70 ± 0.82	0.62 ± 0.07	0.03 ± 0.17	5.13 ± 2.43
	2	1.56 ± 0.59	1.50 ± 0.71	0.55 ± 0.68	1.27 ± 0.92	1.06 ± 0.91	1.05 ± 0.94	0.08 ± 0.27	7.06 ± 3.30
	3	1.45 ± 0.50	1.57 ± 0.80	0.62 ± 0.82	1.50 ± 0.89	1.31 ± 1.00	1.33 ± 0.82	0.07 ± 0.26	7.86 ± 2.98
	4	1.75 ± 0.53	1.71 ± 0.81	0.75 ± 0.79	1.92 ± 0.58	1.58 ± 0.72	1.38 ± 0.71	0.00 ± 0.00	9.08 ± 2.77
	5	2.00 ± 0.00	2.50 ± 0.71	1.50 ± 0.71	2.50 ± 0.71	2.00 ± 0.00	2.00 ± 0.00	0.00 ± 0.00	12.50 ± 2.12
	*F(p)*	5.70* (0.000)	4.32* (0.001)	4.16* (0.001)	7.24* (0.000)	6.89* (0.000)	8.85* (0.000)	1.02 (0.407)	14.83* (0.000)

**Table 2b:** BALI categorical and total scores by Cardiovascular Risk Factors for Old Group (*n* = 97).

BALI category		GMSV	DWM	PV	BG	IT	GA	Others	Total
Hypertension	No (n)	1.41 ± 0.50	1.43 ± 0.75	0.43 ± 0.62	1.04 ± 0.87	1.11 ± 0.95	0.93 ± 0.77	0.04 ± 0.21	6.41 ± 2.56
	Yes (n)	1.86 ± 0.49	1.90 ± 0.57	1.12 ± 0.79	1.86 ± 0.78	1.63 ± 0.80	1.78 ± 0.78	0.06 ± 0.24	10.2 ± 2.67
	*T(p)*	4.48* (0.00)	3.46* (0.001)	4.70* (0.00)	4.91* (0.00)	2.92* (0.004)	5.37* (0.00)	0.34 (0.736)	7.15* (0.00)
Diabetic Mellitus	No	1.61 ± 0.54	1.92 ± 0.49	0.75 ± 0.77	1.38 ± 0.93	1.33 ± 0.92	1.29 ± 0.89	0.06 ± 0.24	8.01 ± 3.19
	Yes	1.92 ± 0.49	2.23 ± 0.44	1.08 ± 0.86	2.08 ± 0.49	1.69 ± 0.75	2.0 ± 0.58	0.0 ± 0.00	11.0 ± 2.08
	*T(p)*	1.99* (0.050)	3.19 (0.002)	1.40 (0.166)	2.63* (0.010)	1.33 (0.185)	2.81 (0.006)	0.90 (0.372)	3.26* (0.002)
Hyperlipidemia	No	1.67 ± 0.54	1.58 ± 0.75	0.70 ± 0.81	1.64 ± 0.90	1.33 ± 0.85	1.48 ± 1.06	0.03 ± 0.17	8.42 ± 3.45
	Yes	1.64 ± 0.55	1.73 ± 0.67	0.84 ± 0.78	1.39 ± 0.92	1.41 ± 0.94	1.32 ± 0.78	0.063 ± 0.24	8.41 ± 3.14
	*T(p)*	0.22 (0.824)	1.06 (0.293)	0.87 (0.389)	1.26 (0.211)	0.37* (0.710)	0.83* (0.411)	0.67* (0.502)	0.03* (0.979)
BMI > 25	No	1.50 ± 0.56	1.50 ± 0.71	0.74 ± 0.71	1.34 ± 0.88	1.12 ± 0.95	1.06 ± 0.69	0.12 ± 0.33	7.35 ± 2.67
	Yes	1.73 ± 0.51	1.78 ± 0.68	0.83 ± 0.83	1.56 ± 0.93	1.52 ± 0.86	1.56 ± 0.93	0.02 ± 0.13	8.98 ± 3.37
	*T(p)*	2.03* (0.045)	1.89 (0.062)	0.53 (0.595)	1.20 (0.235)	2.14* (0.035)	2.19* (0.031)	1.06 (0.290)	2.44* (0.017)
Current Smoker	No	1.67 ± 0.55	1.70 ± 0.68	0.86 ± 0.82	1.45 ± 0.90	1.38 ± 0.91	1.37 ± 0.92	0.07 ± 0.25	8.51 ± 3.27
	Yes	1.58 ± 0.50	1.62 ± 0.77	0.58 ± 0.65	1.54 ± 0.98	1.38 ± 0.92	1.42 ± 0.78	0.0 ± 0.00	8.13 ± 3.15
	*T(p)*	0.69 (0.493)	0.45 (0.657)	1.52 (0.133)	0.41 (0.679)	0.04 (0.968)	0.22 (0.823)	1.32 (0.192)	0.50 (0.618)
Number of CVRFs	0	1.00 ± 0.00	1.00 ± 0.82	0.25 ± 0.50	1.50 ± 1.00	1.00 ± 0.82	0.75 ± 0.50	0.00 ± 0.00	5.50 ± 0.58
	1	1.42 ± 0.51	1.42 ± 0.84	0.53 ± 0.77	0.95 ± 0.78	1.10 ± 0.99	0.74 ± 0.73	0.11 ± 0.32	6.26 ± 2.66
	2	1.76 ± 0.55	1.71 ± 0.58	0.82 ± 0.72	1.50 ± 0.96	1.29 ± 0.91	1.44 ± 0.96	0.09 ± 0.29	8.62 ± 3.23
	3	1.63 ± 0.49	1.67 ± 0.68	0.89 ± 0.89	1.52 ± 0.94	1.44 ± 0.93	1.59 ± 0.80	0.00 ± 0.00	8.74 ± 3.12
	4	1.91 ± 0.54	2.18 ± 0.40	1.00 ± 0.77	2.00 ± 0.45	2.00 ± 0.45	1.91 ± 0.54	0.00 ± 0.00	11.0 ± 1.90
	5	2.00 ± 0.00	2.50 ± 0.71	1.50 ± 0.71	2.50 ± 0.71	2.00 ± 0.00	2.00 ± 0.00	0.00 ± 0.00	12.5 ± 2.12
	*F(p)*	3.14* (0.012)	3.32* (0.008)	1.40 (0.231)	2.74* (0.024)	1.88 (0.106)	4.42* (0.001)	0.87 (0.501)	5.54* (0.000)

**Table 2c:** BALI categorical and total scores by Cardiovascular Risk Factors for Young Group (*n* = 132).

BALI category		GMSV	DWM	PV	BG	IT	GA	Others	Total
Hypertension	No (n)	1.20 ± 0.51	1.14 ± 0.81	0.24 ± 0.43	0.96 ± 0.83	0.63 ± 0.80	0.60 ± 0.60	0.03 ± 0.17	4.8 ± 2.3
	Yes (n)	1.52 ± 0.51	1.33 ± 0.92	0.37 ± 0.63	1.59 ± 0.79	1.11 ± 0.80	0.81 ± 0.62	0.07 ± 0.27	6.8 ± 2.4
	*T(p)*	2.91* (0.004)	1.05 (0.293)	1.29 (0.199)	3.55* (0.000)	2.80* (0.006)	2.80* (0.006)	1.10 (0.273)	4.03* (0.000)
Diabetic Mellitus	no	1.27 ± 0.53	1.16 ± 0.84	0.27 ± 0.48	1.09 ± 0.86	0.72 ± 0.83	0.64 ± 0.61	0.04 ± 0.19	5.2 ± 2.47
	yes	1.00 ± 0.00	2.00 ± 0.00	0.00 ± 0.00	1.30 ± 1.15	1.00 ± 0.00	0.67 ± 0.58	0.00 ± 0.00	6.00 ± 1.00
	*T(p)*	0.89 (0.376)	1.73 (0.087)	0.98 (0.331)	0.49* (0.623)	0.58 (0.562)	0.07 (0.948)	− 0.35 (0.730)	0.56* (0.575)
Hyperlipidemia	no	1.17 ± 0.53	1.10 ± 0.83	0.24 ± 0.43	0.81 ± 0.80	00.47 ± 0.65	0.47 ± 0.50	0.0 ± 0.00	4.26 ± 2.15
	yes	1.34 ± 0.50	1.24 ± 0.84	0.28 ± 0.51	1.31 ± 0.84	0.93 ± 0.88	0.78 ± 0.65	0.068 ± 0.25	5.96 ± 2.42
	*T(p)*	1.82 (0.071)	0.95 (0.343)	0.51 (0.614)	3.45* (0.001)	3.37* (0.001)	3.09* (0.003)	2.03* (0.044)	4.20* (0.00)
BMI > 25	no	1.21 ± 0.53	1.12 ± 0.87	0.21 ± 0.41	1.00 ± 0.82	0.60 ± 0.73	0.53 ± 0.57	0.02 ± 0.13	4.68 ± 2.36
	yes	1.31 ± 0.52	1.23 ± 0.81	0.31 ± 0.52	1.16 ± 0.89	0.83 ± 0.88	0.73 ± 0.62	0.05 ± 0.23	5.61 ± 2.45
	*T(p)*	1.05 (0.297)	0.71 (0.482)	1.15 (0.252)	1.06 (0.292)	1.61 (0.111)	1.96 (0.052)	1.06 (0.290)	2.19* (0.030)
Current Smoker	no	1.26 ± 0.51	1.14 ± 0.84	0.24 ± 0.43	1.02 ± 0.83	0.68 ± 0.76	0.60 ± 0.59	0.03 ± 0.18	4.97 ± 2.28
	yes	1.29 ± 0.57	1.29 ± 0.84	0.32 ± 0.57	1.26 ± 0.92	0.84 ± 0.95	0.76 ± 0.63	0.05 ± 0.23	5.82 ± 2.77
	*T(p)*	0.34 (0.735)	0.94 (0.349)	0.78 (0.440)	1.47 (0.144)	1.02 (0.308)	1.44 (0.152)	0.56 (0.576)	1.82 (0.072)
Number of CVRFs	0	1.11 ± 0.47	1.06 ± 0.94	0.11 ± 0.32	0.78 ± 0.73	0.50 ± 0.71	0.39 ± 0.50	0.00 ± 0.00	3.94 ± 2.31
	1	1.23 ± 0.55	1.08 ± 0.79	0.33 ± 0.47	0.98 ± 0.85	0.56 ± 0.70	0.58 ± 0.54	0.00 ± 0.00	4.75 ± 2.21
	2	1.28 ± 0.52	1.22 ± 0.79	0.16 ± 0.37	0.97 ± 0.82	0.69 ± 0.82	0.59 ± 0.67	0.06 ± 0.25	4.97 ± 2.25
	3	1.24 ± 0.44	1.47 ± 0.94	0.24 ± 0.44	1.41 ± 0.87	1.18 ± 1.07	1.00 ± 0.71	0.18 ± 0.39	6.71 ± 2.20
	4	1.62 ± 0.50	1.31 ± 0.85	0.54 ± 0.78	1.85 ± 0.69	1.23 ± 0.73	0.92 ± 0.49	0.00 ± 0.00	7.46 ± 2.33
	5	-	-	-	-	-	-	-	-
	*F(p)*	1.99 (0.100)	0.90 (0.466)	3.84 (0.428)	4.50* (0.002)	3.70* (0.007)	3.39* (0.011)	1.39 (0.847)	7.22* (0.000)

Note: data are presented as mean ± standard deviation unless specified otherwise. GM-SV, gray matter and subcortical lesions—subcortical dilated perivascular spaces; DWM, deep white matter lesions; PV, periventricular white matter lesions; BG, lesions in the basal ganglia and surrounding areas; IT, lesions in the infratentorial regions; GA, global atrophy. BALI, Brain Atrophy and Lesion Index; CVRF, cardiovascular risk factors; T (p): Student *T*-test comparing the groups with vs. without a given risk factor (level of significance); F (p): ANOVA test comparing groups with the different number of risk factor (level of significance). *p < 0.05.

**Figure 3:**
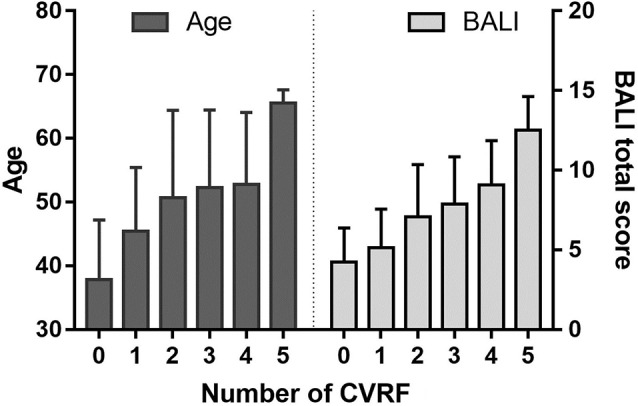
Age and the BALI as a function of the cardiovascular risk factors (CVRFs). Data are presented as mean ± standard variation of age (left panel) and the BALI total score (right panel) for the subjects with each number of CVRF.

**Figure 4:**
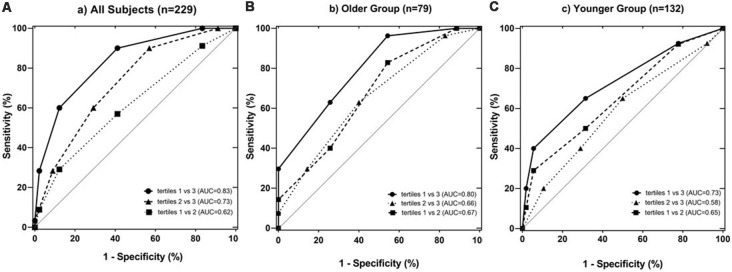
Receiver operating characteristic (ROC) analysis testing the identification of individuals with different levels of brain structural changes based on the tertiled BALI scores, by the number of CVRF *per se*. Analyses were performed for all subjects in the sample (**A**; *n* = 229), and separately for the stratified older (**B**; *n* = 97; age ≥ 50 years) and younger (**C**; *n* = 132; age < 50 years) groups. The differentiation performance was estimated using the area under the ROC curve (AUC). The lower and upper bounds and level of significance were also provided for **(A–C**; see below). In each sub-figure, square and dotted line: tertiles 1 vs. 2; circles and solid line: tertiles 1 vs. 3; triangles and dashed line: tertiles 2 vs. 3; the gray diagonal line: indicator of AUC = 0.50.

Combining the CVRF, the mean BALI total score was greater as the number of CVRF increased (*F* = 14.83, *p* < 0.001; Table 2a; Figure 3); this relationship appeared to be stronger than the increase in the BALI score with age. The prediction using number of CVRF resulted in an AUC = 0.832 (0.767–0.898, *p* < 0.001) for differentiating individuals in the tertiles with low vs. high BALI scores (Figure 4A; Supplementary Table S1). The AUC was 0.797 (0.689–0.905, *p* < 0.001) and 0.728 (0.622–0.833, *p* < 0.001), respectively when the younger and older subgroups were separately analyzed (Figures 4B,C; Supplementary Tables S2, S3). The performance was significant also in differentiation, the BALI-based conjunctive tertiles (e.g., tertiles 1 vs. 2 and tertiles 2 vs. 3 saving younger adults with BALI scores >5), using number of CVRF (Figures 4A–C; Supplementary Tables S1–S3).

Multinomial analyses adjusting for confounders demonstrated an independent association of age and the number CVRF on the BALI. For each year of age advancement, RRR = 1.20, 95% CI = 1.11–1.29 in respect of the low vs. high tertiles; similarly, for any additional CVRF, RRR = 3.63, 95% CI = 2.12–6.23 (Table 3a-1). Regarding the individual exposures, hypertension most significantly increased the risk of having a higher BALI (Table 3a-2). Similar results were yielded when the analysis was repeated using either the younger or the older subgroups, even though the level of significance with detailed comparisons varied (Tables s 3b,c). Notably, certain interesting patterns were only seen in the younger group. For example, the exposures were significant individually and in combination in the older group; in contrast, only the number of CVRF differentiated the BALI status in the younger group (Tables s 3b,c), suggesting the role of deficit accumulation in early detection of brain changes.

**Table 3a:** Multinomial logistic regression models showing the association of the Brain Atrophy and Lesion Index level (by tertiles) with age, sex, education, marriage, work and cardiovascular risk factors (CVRF) for All (*n* = 229).

Tertiles	Factors	Beta	Wald	RRR	95%CI	*p*
**Table 3a-1: Age, sex, education, marriage, work and number of CVRF**						
2 vs. 1	Age	0.09	16.30	1.09	1.05–1.13	0.000*
	Sex (male)	−0.32	0.64	0.73	0.33–1.60	0.425
	Education (college or university)	0.10	0.02	1.10	0.32–3.80	0.881
	Marriage (single)	0.14	0.03	1.15	0.23–5.68	0.860
	Work (retired)	0.55	1.31	1.74	0.67–4.50	0.253
	MR field strength (3.0T)	0.35	0.81	1.41	0.67–3.01	0.367
	Number of CVRF	0.26	2.07	1.30	0.91–1.84	0.150
3 vs. 1	Age	0.18	24.00	1.20	1.11–1.29	0.000*
	Sex(male)	−0.08	0.02	0.92	0.24–3.48	0.902
	Education (college or university)	1.04	1.28	2.82	0.47–16.9	0.257
	Marriage (single)	13.50	<0.00	709144.3	0-0.0	0.983
	Work (retired)	−0.64	0.78	0.53	0.13–2.20	0.378
	MR field strength (3.0T)	1.09	3.11	2.98	0.89–10.05	0.078
	Number of CVRF	1.29	21.90	3.63	2.12–6.23	0.000*
3 vs. 2	Age	0.10	7.71	1.10	1.03–1.18	0.006*
	Sex(male)	0.24	0.14	1.27	0.36–4.49	0.712
	Education (college or university)	0.94	1.31	2.56	0.51–12.82	0.252
	Marriage (single)	13.33	<0.00	614533.0	0-0.0	0.983
	Work (retired)	−1.20	3.26	0.30	0.08–1.11	0.071
	MR field strength (3.0T)	0.75	1.69	2.11	0.69–6.49	0.193
	Number of CVRF	1.03	16.82	2.80	1.71–4.59	0.000*
**Table 3a-2: Age, sex, education, marriage, work and individual CVRF items**						
2 vs. 1	Age	0.09	25.82	1.09	1.05–1.13	0.000*
	Sex (male)	−0.31	1.23	0.74	0.33–1.66	0.461
	Education (college or university)	0.08	0.09	1.08	0.31–3.76	0.903
	Marriage (single)	0.07	0.05	1.07	0.21–5.38	0.934
	Work (retired)	0.60	0.04	1.83	0.69–4.82	0.225
	MR field strength (3.0T)	0.36	4.76	1.44	0.67–3.08	0.353
	Hypertension	0.31	4.26	1.37	0.56–3.33	0.493
	Diabetic Mellitus	0.86	1.39	2.37	0.21–27.15	0.487
	Hyperlipidemia	0.19	2.78	1.21	0.59–2.47	0.593
	BMI > 25	0.36	1.32	1.44	0.71–2.91	0.317
	Current Smoker	0.17	0.31	1.19	0.53–2.68	0.680
3 vs. 1	Age	0.18	22.72	1.20	1.11–1.29	0.000*
	Sex (male)	−0.66	0.79	0.52	0.12–2.22	0.375
	Education (college or university)	1.44	1.94	4.23	0.56–32.03	0.163
	Marriage (single)	13.64	<0.00	839247.5	0-0.0	0.987
	Work (retired)	−0.81	1.11	0.44	0.10–2.01	0.291
	MR field strength (3.0T)	0.97	2.12	2.64	0.72–9.73	0.145
	Hypertension	2.01	12.36	7.50	2.44–23.06	0.000*
	Diabetic Mellitus	2.98	4.11	19.62	1.10–349.06	0.043*
	Hyperlipidemia	2.18	9.02	8.84	2.13–36.66	0.003*
	BMI > 25	1.10	3.23	3.00	0.91–9.97	0.072
	Current Smoker	0.013	<0.00	1.01	0.31–3.33	0.983
3 vs. 2	Age	0.09	6.88	1.10	1.02–1.17	0.009*
	Sex (male)	−0.35	0.25	0.70	0.18–2.81	0.618
	Education (college or university)	1.36	2.11	3.91	0.62–24.68	0.147
	Marriage (single)	13.57	<0.00	783849.2	0-0.0	0.987
	Work (retired)	−1.41	4.17	0.24	0.06–0.95	0.041*
	MR field strength (3.0T)	0.61	0.94	1.84	0.54–6.27	0.332
	Hypertension	1.70	11.67	5.49	2.07–14.60	0.001*
	Diabetic Mellitus	2.11	3.91	8.27	1.02–67.16	0.048*
	Hyperlipidemia	1.98	8.47	7.28	1.91–27.75	0.004*
	BMI > 25	0.74	1.63	2.09	0.67–6.50	0.201
	Current Smoker	−0.16	0.08	0.85	0.29–2.48	0.772

Tertile-all (1): BALI 0–5; Tertile-all (2): BALI 6–8; Tertile-all (3): BALI 9–16. *Indicates p < 0.05.

**Table 3b:** Multinomial logistic regression models showing the association of the Brain Atrophy and Lesion index level (by tertiles) with age, sex, education, marriage, work and cardiovascular risk factors (CVRF) for Old Group (*n* = 97).

Tertiles	Factors	Beta	Wald	RRR	95%CI	*p*
**Table 3b-1: Age, sex, education, marriage, work and number of CVRF**						
2 vs. 1	Age	0.12	1.79	1.13	0.94–1.35	0.181
	Sex (male)	−0.92	1.00	0.40	0.07–2.43	0.318
	Education (college or university)	−16.85	<0.00	4.79e-08	0-0.0	0.991
	Marriage (single)	−1.30	0.49	0.27	0.01–10.49	0.485
	Work (retired)	−1.12	0.57	0.33	0.02–6.02	0.452
	MR field strength (3.0T)	0.83	1.42	2.29	0.59–8.93	0.234
	Number of CVRF	0.75	4.56	2.12	1.06–4.22	0.033
3 vs. 1	Age	0.16	2.34	1.17	0.96–1.44	0.126
	Sex(male)	−1.20	1.09	0.30	0.03–2.85	0.296
	Education (college or university)	−16.79	<0.00	5.09e-08	0-0.0	0.992
	Marriage (single)	12.68	<0.00	321480.3	0-0.0	0.998
	Work (retired)	−3.44	3.88	0.03	0.00–0.98	0.049*
	MR field strength (3.0T)	0.21	0.04	1.24	0.17–8.88	0.832
	Number of CVRF	1.45	9.27	2.03	1.68–10.83	0.002*
3 vs. 2	Age	0.04	0.37	1.04	0.92–1.17	0.541
	Sex(male)	−0.27	0.12	0.76	0.16–3.61	0.730
	Education (college or university)	0.06	<0.00	1.06	0.18–6.13	0.947
	Marriage (single)	13.98	<0.00	1181587.0	0-0.0	0.997
	Work (retired)	−2.32	4.21	0.10	0.01–0.90	0.040*
	MR field strength (3.0T)	−0.61	0.48	0.54	0.10–3.07	0.488
	Number of CVRF	0.70	3.69	2.01	0.99–4.09	0.055
**Table 3b-2: Age, sex, education, marriage, work and individual CVRF items**						
2 vs. 1	Age	0.12	1.63	1.13	0.94–1.36	0.202
	Sex (male)	−1.19	1.44	0.30	0.04–2.13	0.230
	Education (college or university)	−16.81	<0.00	4.98e-08	0-0.0	0.992
	Marriage (single)	−1.66	0.80	0.19	0.01–7.15	0.370
	Work (retired)	−1.03	0.45	0.36	0.02–7.27	0.502
	MR field strength (3.0T)	0.80	0.98	2.22	0.46–10.83	0.322
	Hypertension	1.49	4.62	4.44	1.14–17.33	0.032*
	Diabetic Mellitus	17.05	<0.00	2.54e+07	0-0.0	0.992
	Hyperlipidemia	0.93	1.52	2.55	0.58–11.23	0.217
	BMI > 25	0.27	0.14	1.32	0.32–5.48	0.706
	Current Smoker	0.14	0.03	1.15	0.27–4.84	0.853
3 vs. 1	Age	0.15	1.75	1.16	0.93–1.44	0.186
	Sex (male)	−2.36	3.16	0.95	0.01–1.28	0.076
	Education (college or university)	−17.17	<0.00	3.51e-08	0-0.0	0.992
	Marriage (single)	10.67	<0.00	43090.3	0-0.0	0.998
	Work (retired)	−03.86	4.38	0.02	0.00–0.78	0.036*
	MR field strength (3.0T)	−0.08	0.01	0.92	0.10–8.76	0.942
	Hypertension	3.55	10.40	34.91	4.03–302.45	0.001*
	Diabetic Mellitus	18.17	<0.00	7.78e+07	0-0.0	0.992
	Hyperlipidemia	1.06	1.09	2.89	0.39–21.28	0.297
	BMI > 25	0.02	<0.00	1.02	0.12–8.99	0.985
	Current Smoker	0.31	0.08	1.36	0.15–12.10	0.783
3 vs. 2	Age	0.03	0.16	1.03	0.90–1.17	0.693
	Sex (male)	−1.17	1.46	0.31	0.05–2.06	0.227
	Education (college or university)	−0.35	0.11	0.70	0.09–5.63	0.741
	Marriage (single)	12.33	<0.00	226028.9	0-0.0	0.998
	Work (retired)	−2.83	5.51	0.06	0.01–0.63	0.019*
	MR field strength (3.0T)	−0.88	0.84	0.41	0.06–2.73	0.359
	Hypertension	2.06	4.90	7.86	1.27–48.77	0.027*
	Diabetic Mellitus	1.12	1.53	3.06	0.52–18.05	0.216
	Hyperlipidemia	0.13	0.03	1.14	0.25–5.14	0.869
	BMI > 25	−0.25	0.07	0.78	0.13–4.79	0.785
	Current Smoker	0.17	0.03	1.19	0.19–7.41	0.855

Tertile-old (1): BALI 2–7; Tertile-old (2): BALI 8–10; Tertile-old (3): BALI 11–16. *Indicates p < 0.05.

**Table 3c:** Multinomial logistic regression models showing the association of the Brain Atrophy and Lesion Index level (by tertiles) with age, sex, education, marriage, work and cardiovascular risk factors (CVRF) for Young Group (*n* = 132).

Tertiles	Factors	Beta	Wald	RRR	95%CI	*p*
**Table 3c-1: Age, sex, education, marriage, work and number of CVRF**						
2 vs. 1	Age	0.05	1.42	1.05	0.97–1.13	0.233
	Sex (male)	−0.68	1.53	0.50	0.17–1.49	0.216
	Education (college or university)	−0.50	0.31	0.61	0.10–3.55	0.580
	Marriage (single)	−0.28	0.10	0.75	0.13–4.34	0.751
	Work (retired)	0.09	0.02	1.10	0.25–4.83	0.902
	MR field strength (3.0T)	−0.27	0.28	0.76	0.28–2.07	0.596
	Number of CVRF	0.46	3.62	1.59	0.99–2.55	0.057
3 vs. 1	Age	0.19	14.88	1.21	1.10–1.33	0.00*
	Sex (male)	−0.86	1.85	0.42	0.12–1.46	0.174
	Education (college or university)	0.63	0.35	1.87	0.23–15.0	0.556
	Marriage (single)	14.94	<0.00	3064624.0	0-0.0	0.992
	Work (retired)	1.01	1.52	2.73	0.55–13.52	0.218
	MR field strength (3.0T)	0.72	1.39	2.05	0.62–6.75	0.239
	Number of CVRF	0.59	5.06	1.80	1.08–2.99	0.024*
3 vs. 2	Age	0.14	14.88	1.15	1.05–1.27	0.003*
	Sex(male)	−0.17	1.85	0.84	0.22–3.28	0.805
	Education (college or university)	1.12	0.35	3.08	0.47–20.27	0.242
	Marriage (single)	15.21	<0.00	4067714.0	0-0.0	0.992
	Work (retired)	0.99	1.52	2.49	0.59–10.48	0.214
	MR field strength (3.0T)	0.12	1.39	2.68	0.85–8.50	0.094
	Number of CVRF	−24.87	5.06	1.13	0.71–1.80	0.601
**Table 3c-2: Age, sex, education, marriage, work and individual CVRF items**						
2 vs. 1	Age	0.05	1.21	1.05	0.97–1.13	0.000*
	Sex (male)	−0.70	1.53	0.50	0.16–1.51	0.142
	Education (college or university)	−0.68	0.55	0.51	0.09–3.02	0.651
	Marriage (single)	−0.29	0.10	0.75	0.12–4.55	0.991
	Work (retired)	0.20	0.07	1.22	0.27–5.43	0.236
	MR field strength (3.0T)	−0.24	0.22	0.79	0.29–2.16	0.266
	Hypertension	1.10	2.42	3.02	0.75–12.12	0.239
	Diabetic Mellitus	16.59	<0.00	3.99e+10	0-0.0	0.995
	Hyperlipidemia	0.37	0.57	0.71	0.55–3.80	0.113
	BMI > 25	0.00	<0.00	0.48	0.39–2.56	0.371
	Current Smoker	0.15	0.07	0.68	0.37–3.68	0.817
3 vs. 1	Age	0.19	13.95	1.21	1.10–1.34	0.000*
	Sex (male)	−0.97	2.15	0.38	0.10–1.39	0.142
	Education (college or university)	0.48	0.20	1.62	0.20–13.06	0.651
	Marriage (single)	14.65	<0.00	2305598.0	0-0.0	0.991
	Work (retired)	0.97	1.41	2.64	0.53–13.12	0.236
	MR field strength (3.0T)	0.68	1.24	1.97	0.60–6.48	0.266
	Hypertension	0.89	1.39	2.45	0.55–10.85	0.239
	Diabetic Mellitus	15.80	<0.00	7257029.0	0-0.0	0.995
	Hyperlipidemia	0.91	2.51	2.49	0.80–7.72	0.113
	BMI > 25	0.50	0.80	1.65	0.55–4.93	0.371
	Current Smoker	0.15	0.05	1.16	0.33–4.07	0.817
3 vs. 2	Age	0.15	8.44	1.16	1.05–1.28	0.004*
	Sex (male)	−0.27	0.14	0.76	0.19–3.13	0.705
	Education (college or university)	1.16	1.43	3.18	0.48–21.30	0.232
	Marriage (single)	14.94	<0.00	3088895.0	0-0.0	0.990
	Work (retired)	0.77	1.04	2.16	0.49–9.52	0.309
	MR field strength (3.0T)	0.92	2.40	2.50	0.78–7.97	0.121
	Hypertension	−0.21	0.11	0.81	0.23–2.86	0.745
	Diabetic Mellitus	−0.79	0.34	0.45	0.03–6.59	0.562
	Hyperlipidemia	0.54	0.80	1.72	0.52–5.65	0.372
	BMI > 25	0.50	0.77	1.65	0.54–5.02	0.381
	Current Smoker	−0.00	<0.00	1.82e-11	0.30–3.29	0.997

Tertile-young (1): BALI 0–4; Tertile-young (2): BALI 5–6; Tertile-young (3): BALI 7–11. *Indicates p < 0.05.

## Discussion

In this study, we investigated whole-brain structural changes on MRI and their association with cardiovascular risks, individually and in combination using number of the exposures, in a sample of healthy adults aged 24–80 years undergoing routine health evaluations. Brain structure status was evaluated using the BALI on T2WI. We identified multiple lesions across the age range including younger adults and found an independent association of the BALI with age and the number of CVRFs. The study contributed to the current literature by extending the association between BALI and age to a group of subjects with a wide age range and by demonstrating an independent association between number of vascular risk factors and BALI score in a multivariate model adjusted for demographics. The study has implications in understanding the effects of multiple coexisting risk factors on the brain at a young age, a key for developing early preventive strategies.

The study used MRI evaluation of BALI as a proxy measure of whole-brain structural health status. In contrast to traditional MRI based assessments that mostly focus on understanding one specific type of deficit at a time (Fazekas et al., 1987; Scheltens et al., 1993; Hogstrom et al., 2013; Ghadery et al., 2015), the BALI takes an holistic approach to evaluating the brain as a functioning system, by assessing and integrating several changes commonly seen in the aging brain, including GA, changes in both supratentorial and infratentorial white matter and small vessels (Zhang et al., 2011; Guo et al., 2014a,b, 2017). This undertaking is a response to the pressing demand to better understand the brain as a complex system: multiple factors can contribute to brain structural changes of various types, in a similar way as they do to that of general health and cognition (Rockwood and Mitnitski, 2007; Song et al., 2011; Mitnitski et al., 2013; Armstrong et al., 2016; de Beer and Scheltens, 2016). Such whole-brain approaches are also shown to be crucial in understanding the topological properties of brain function in the aging brain (Geib et al., 2017).

Emphasizing the role of a summary quantitative assessment of subtle brain changes in an aging-oriented perspective is also of clear clinical significance. The existing geriatric literature has already identified the prognostic role of subtle neurological signs (Ferrucci et al., 2004; Inzitari et al., 2008). In all studies to date, age-specific mean BALI scores increase with age in older adults, either linearly or exponentially (Song et al., 2013; Guo et al., 2014b; Grajauskas et al., 2019). This confirms the value of simultaneous assessment of multiple brain structural changes, and facilitates a challenging aspect of understanding brain aging, which is that such lesions tend to co-occur (Chen et al., 2011; Zhang et al., 2012; Park et al., 2013; Song et al., 2013; Tosto et al., 2014; Cai et al., 2015; Smith and Beaudin, 2018). Despite general co-occurrence, various lesions signify various insults, so that the BALI scores have demonstrable validity and reliability in aiding dementia diagnosis [e.g., normal aging, mild cognitive impairment (MCI), Alzheimer’s disease (AD)] and in predicting individuals at greater (age-adjusted) risk of converting from MCI to AD (Zhang et al., 2012; Song et al., 2013) relationship between age. Here, by also studying younger subjects, we extend the findings of the whole brain structural health changes across the adult life course, demonstrating that accumulation of the structural deficits in the brain can begin in young adulthood. This appears to have consequences. For example, for most BALI categories, a higher rate and a greater mean subscore were observed at a higher age on average (Table 1a; Figure 2; Supplementary Figures S1–S3), suggesting that brain structural problems become more common and more severe with age. “A diversity of factors subtly affect brain integrity, but it may not be until late adulthood that the aggregation of these insults exert a measurable effect on cognition.” (Reuter-Lorenz and Park, 2014). By stratifying the younger and older groups and demonstrating the risks in both groups, our data evidence the need to effectively capture the subtle changes early in adulthood to inform the development of early preventive strategies.

Here too, different problems also showed varied age-associated change patterns. In this sample of healthy people from China, small vessel problems were already quite prevalent as early as age 30. Of interest, in the face of a higher number of CVRF, GA becomes more common and more marked with age, whereas periventricular deficits maintained relatively stable until age 55+ years (Figures 2, 3). Of note, in other settings, when these age-associated changes co-exist, they further worsen brain health and impair cognitive and other brain functions (Ashford et al., 2011; Song et al., 2013; Duering et al., 2014; Boulouis et al., 2017); e.g., a recent study using 3T MRI has characterized the effect of microinfarcts on cognitive impairment in cerebrovascular disease (Hilal et al., 2016).

The independent effects of age and cardiovascular risk exposures confirm that age *per se* is not the only factor affecting brain structural health. Older subjects also tend to accumulate more CVRFs on average (Figure 3, left panel). Even so, it may not just be the aggregation of the CVRF affected brain structural health. Similar relationships between age and cardiovascular conditions have been reported by numerous previous reports (Luchsinger et al., 2005), e.g., hypertension (Gerhard et al., 1996), heart disease (Izzo et al., 2018), diabetes (Huang et al., 2014), smoking (Anstey et al., 2007), and BMI (Anstey et al., 2011), such that when combined, deficits in aggregation are strongly associated with overall health (Song et al., 2005; Mitnitski et al., 2007), even when age and several other demographical factors were adjusted, an addition of any cardiovascular risk exposure increased the relative risk of having a worse brain health condition by as high as 260% (Table 3a-1). It is worth emphasizing that, especially in younger adults, the emerging impact of the brain health risk might have been undetected if the exposures were studied only individually, whereas the impact of multiple statistically small impact was revealed by the summarizing CVRF score (Table 3c-1 vs. Table 3c-2).

Our data must be interpreted with caution. First, despite the power of the CVRF, they are not the only deficits to accumulate with age (Rockwood and Mitnitski, 2007; Rockwood et al., 2017). A range of health deficits can accumulate with age to affect the risk of cognitive impairment, including dementia (Song et al., 2011, 2014; Sterniczuk et al., 2013; Searle and Rockwood, 2015; Canevelli et al., 2017). Here, we had access to complete data only on these five risk factors. Future studies are needed to test the role of genetic risk factors (de Frias et al., 2014; Hickman et al., 2016) and expand the range of age-related health deficits, whether recognized as traditional risk factors for brain structural lesions or not. As a first approximation, in any model in which chronological age is importantly related to an adverse outcome, heterogeneity in that risk will be lessened by considering other age-related health deficits, which can serve as a proxy for biological age (Mitnitski et al., 2013, 2017).

Second, and perhaps the main limitation of the study concerns the uncertain external validity of data, as the sample not only included a relatively limited convenience sample of subjects in good health but also subjects with a high education level from a single ethnic group—which is known to be particularly liable to cerebrovascular damage in comparison with Western populations. The impact of the accumulation of cardiovascular risks on brain health has not been well studied for them; our data contributed to the understanding of the extent of the impact at younger and older ages. The extent to which the findings can be generalized to a general population calls for future population-based research with larger samples. At present, large-scale brain imaging studies have mostly enrolled only older adults, with middle-aged adults (e.g., 45 years) being about the youngest (Cox et al., 2016). The lack of larger dataset involving younger adults has limited a possibly more representative investigation for us to better tackle the early occurrence of multiple brain changes. Given the increasing importance paid to midlife risks in relation to late-life dementia (Livingston et al., 2017; Hou et al., 2018), more work on brain structural changes would be useful.

Third, subjects in our dataset were mostly in good health, undergoing an annual health examination. The data did not contain information on cognition, other than excluding those with cognitive impairment diagnosis. Other limitations relate to MRI evaluation and processing. Although most of the MRI scans used 3.0T, the other scans (i.e., 26.2%) were acquired using a conventional 1.5T MRI. Given that high field strength can allow high signal-to-noise ratios and greater image contrast, it is not surprising that a significantly higher mean BALI total scores using 3.0T over 1.5T images (6.84 ± 3.28 vs.5.80 ± 2.92, *t* = 2.17, *p* = 0.031). On the other hand, the evaluation has been proven reliable (Gu et al., 2018) and data collection was not biased for field-strength, in terms of age and possession of the risk factors under study (e.g., mean age 48.77 ± 12.87 vs. 47.00 ± 11.41, *t* = 0.94, *p* = 0.348; mean number of risk factors 1.93 ± 1.18 vs. 1.78 ± 1.24, *t* = 0.85, *p* = 0.398). Previous studies have also demonstrated the reliability and comparability of both field strengths (Guo et al., 2014b; Gu et al., 2018).

Finally, we used 2D T2-weighted MRI in BALI evaluation. T2WI is known to be sensitive in revealing pathological changes (Nadgir and Yousem, 2017) and has been validated for use in BALI studies (Guo et al., 2014a,b). Although it is well known that T2WI is associated with a greater sensitivity, particularly in identifying subtle changes of the imaging-contrast-reliant categories (e.g., GM-SV and DMW), in clinical settings the slice thickness for T2WI is typically 5.0 mm (as the case with our study). This is larger than that for 3D T1WI (e.g., 1 mm—higher spatial resolution) which has excellent brain tissue contrast and great spatial resolution and is widely used in research. The 2D T2WI is a routine clinical sequence whereas 3D T1WI is not, presumably due to scan time. Previous studies comparing the two image sequences in BALI evaluation have revealed close correlations. T2WI-based evaluation can help enhance feasibility for potential clinical translation of the BALI method when it is infeasible to apply algorithms for optimized sensitivity of BALI evaluation due to unavailability of multiple sequences (Guo et al., 2017; Nadgir and Yousem, 2017).

Also related to the potential clinical application of BALI, we evaluated the BALI using the AccuImage software package, which takes DICOM data as input images, rather than using the MRICRO as recommended by previous studies, which takes ANALYZE or NIFTI format. Given that DICOM is the default format with all MRI scanners, brain scans can be readily evaluated without the need for reformatting. Despite these limitations, in this study, the evaluation of BALI strictly followed standard procedures (Guo et al., 2014a) and, as demonstrated elsewhere, the resulting BALI scores showed great intra-rater and inter-rater reliability (Gu et al., 2018).

In conclusion, brain structural lesions were evaluated collectively in a convenience sample of 229 adults from ages 24–80. The summative BALI scores consistently captured deficits in brain atrophy and lesion subtypes, reflecting that the burden of age and risk factors on brain health can begin at even younger adulthood. CVRF and age showed independent effects on the accumulation of brain structural deficits, providing an explanation of brain health changes beyond the effect of age. Further research with larger population samples is needed to verify the study finding and to understand the extent to which other age-related health deficits can increase the risk of abnormalities in brain structure and function.

## Ethics Statement

This is an exploratory analysis of routinely collected health evaluation data, for which all the data had been anonymized with de-identification before retrieval for analysis. The research protocol received approvals from the Research Ethics Committees of Beijing Hospital (2014-4-4052). Additional approval for secondary MRI analysis was received from the Fraser Health Research Ethics Board (FHREB2015-030).

## Author Contributions

TG, MC, KR and XS conceived and designed the experiments. TG, HG and XS performed the experiments and MRI evaluation, analyzed the data and prepared the result. MZ reviewed and finalized the analyses. CF and ZS contributed materials. TG and XS drafted the first manuscript. KR and XS revised the manuscript. All authors reviewed, edited and approved the submission.

## Conflict of Interest Statement

TG receives research support from the Capital’s Funds for Health Improvement and Research of China and a fellowship award from Beijing Hospital to conduct postdoctoral research in Canada. CF reports no disclosures. ZS reports no disclosures. HG receives a fellowship award from the China Scholarship Council to conduct postdoctoral research in Canada. MZ reports no disclosures. MC reports no disclosures. KR receives career support from the Dalhousie Medical Research Foundation as the Kathryn Allen Weldon Professor of Alzheimer Research, and research support from the Canadian Institutes of Health Research, the Nova Scotia Health Research Foundation, the Capital Health Research Fund and the Fountain Family Innovation Fund of the Nova Scotia Health Authority Foundation. He is Associate Director of the Canadian Consortium on Neurodegeneration in Aging, which is funded by the CIHR, with additional funding from the Alzheimer Society of Canada and several other charities, as well as from Pfizer Canada and Sanofi Canada. He is Founder, President and Chief Science Officer of DGI Clinical, which has contracts with pharma on individualized outcome measurement. In 2017 he attended an advisory board meeting with Lundbeck. Otherwise all personal fees are for invited guest lectures and academic symposia. XS received operating research grant support from the Canadian Institutes of Health Research, Surrey Hospital & Outpatient Centre Foundation, Michael Smith Foundation of Health Research, and the Canadian Frailty Network.

## Supplementary Material

FIGURE S1 The Brain Atrophy and Lesion Index (BALI) subscores by age group. Data are presented as the percentage of people with each subscore (Cx) for each age group. Circles: Cx = 0; squares: Cx = 1; up-triangles: Cx = 2; down-triangles: Cx = 3 or more. GM-SV, gray matter and subcortical lesionssubcortical dilated perivascular spaces; DWM, deep white matter lesions; PV, periventricular white matter lesions; BG, lesions in the basal ganglia and surrounding areas; IT, lesions in the infratentorial regions; GA, global atrophy.

FIGURE S2 Number and percentage present of subjects in each age group as grouped by BALI subscores. Data are presented as the number of subjects in each age group (#) and percentage of subjects in each age group (%). For the subscore, outcome of *χ*^2^ analysis testing the trend of the age and subscore association is provided, together with the level of significance, p. GM-SV, gray matter and subcortical lesions- subcortical dilated perivascular spaces; DWM, deep white matter lesions; PV, periventricular white matter lesions; BG, lesions in the basal ganglia and surrounding areas; IT, lesions in the infratentorial regions; GA, global atrophy.

FIGURE S3 The Brain Atrophy and Lesion Index (BALI) subscores by age group. Data are presented as mean ± standard deviation of the subscores of the BALI for each age group. GM-SV, gray matter and subcortical lesions—subcortical dilated perivascular spaces; DWM, deep white matter lesions; PV, periventricular white matter lesions; BG, lesions in the basal ganglia and surrounding areas; IT, lesions in the infratentorial regions; GA, global atrophy.

TABLE S1 Figure 4A ROC for All subjects (*n* = 229). Tertile (1): BALI 0-5; Tertile (2): BALI 6-8; Tertile (3): BALI 9-16. **p* < 0.05.

TABLE S2 Figure 4B ROC for Older Group (*n* = 97). Tertile (1): BALI 2-7; Tertile (2): BALI 8-10; Tertile (3): BALI 11-16. **p* < 0.05.

TABLE S3 Figure 4C ROC for Younger Group (*n* = 132). Tertile (1): BALI 0-4; Tertile (2): BALI 5-6; Tertile (3): BALI 7-11. **p* < 0.05.
