# Supplementary figures and images for: Age-Related Whole-Brain Structural Changes in Relation to Cardiovascular Risks Across the Adult Age Spectrum
Source: Front Aging Neurosci. 2019 Apr 24;11:85. doi: 10.3389/fnagi.2019.00085 (PMC6492052; doi:10.3389/fnagi.2019.00085)

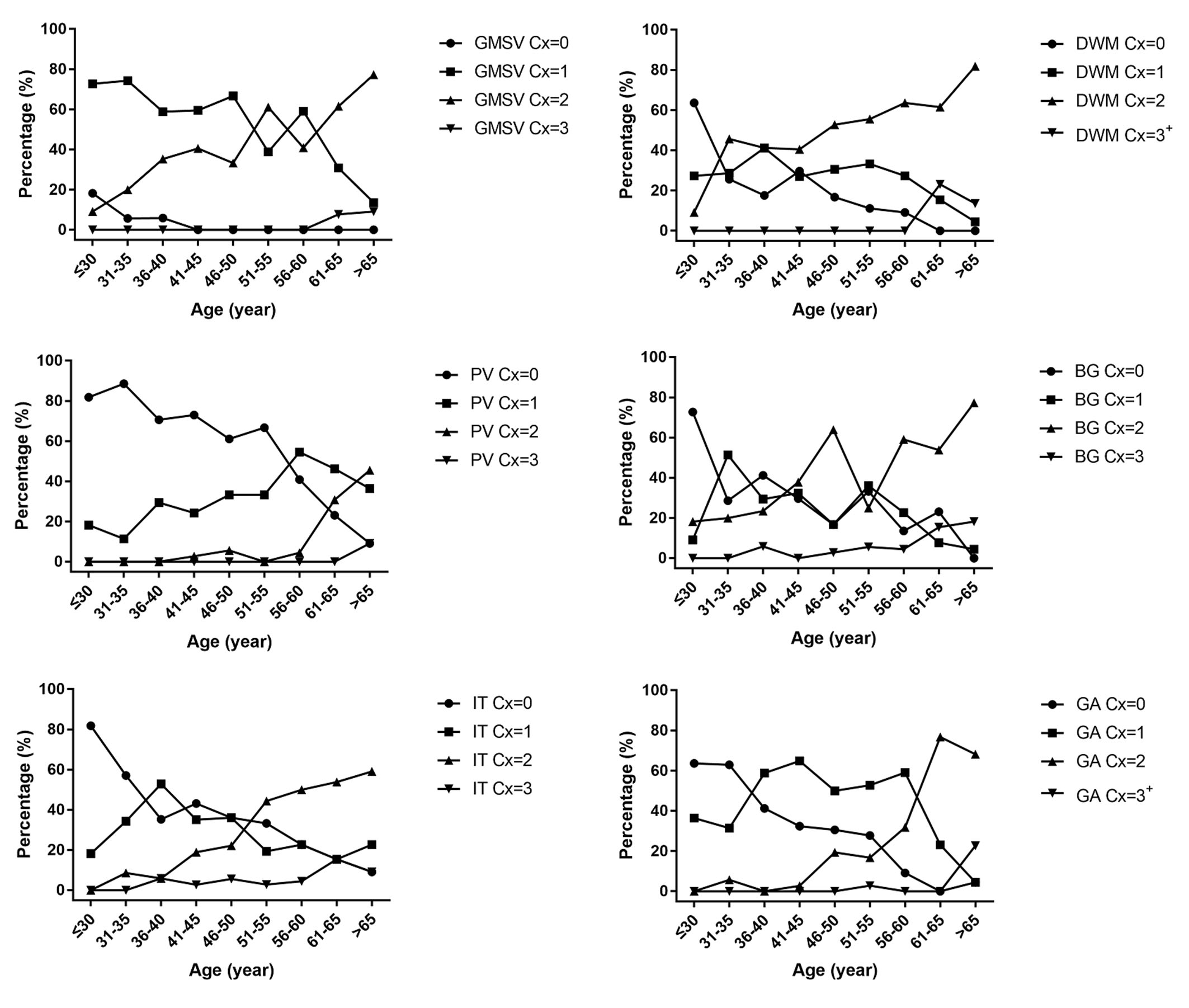

Supplement: FIGURE S1 — The Brain Atrophy and Lesion Index (BALI) subscores by age group. Data are presented as the percentage of people with each subscore (Cx) for each age group. Circles: Cx = 0; squares: Cx = 1; up-triangles: Cx = 2; down-triangles: Cx = 3 or more. GM-SV, gray matter and subcortical lesionssubcortical dilated perivascular spaces; DWM, deep white matter lesions; PV, periventricular white matter lesions; BG, lesions in the basal ganglia and surrounding areas; IT, lesions in the infratentorial regions; GA, global atrophy. [file Image_1.JPEG]

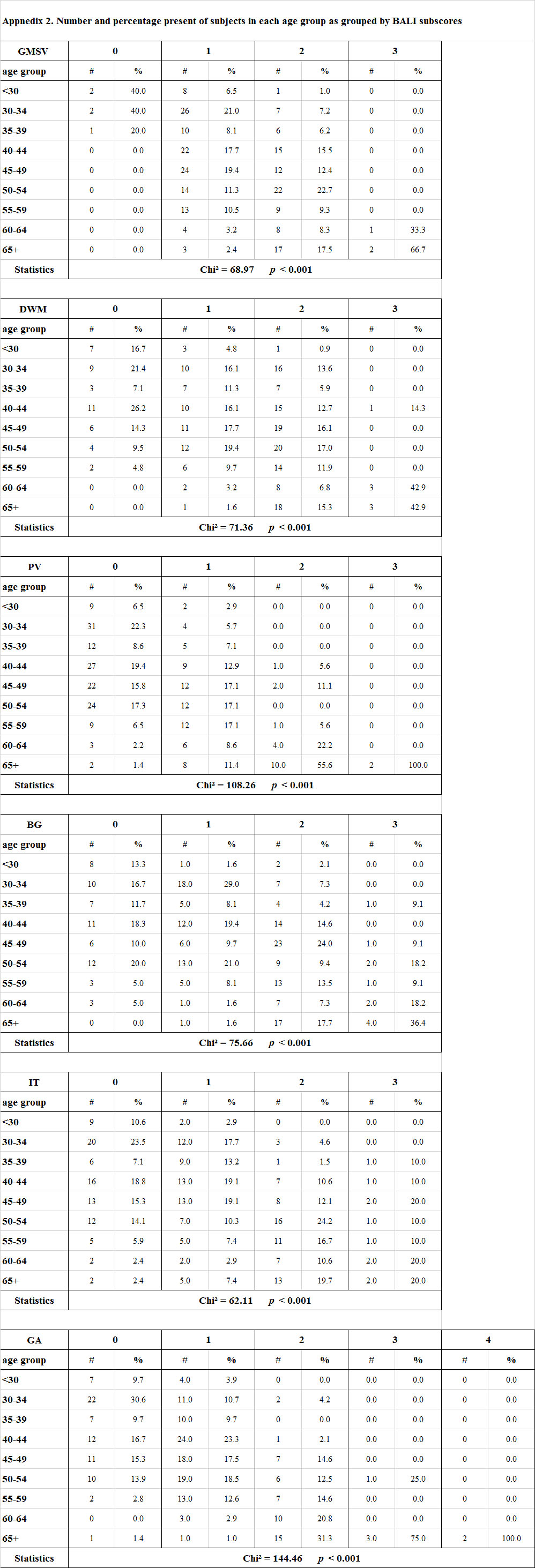

Supplement: FIGURE S2 — Number and percentage present of subjects in each age group as grouped by BALI subscores. Data are presented as the number of subjects in each age group (#) and percentage of subjects in each age group (%). For the subscore, outcome of χ2 analysis testing the trend of the age and subscore association is provided, together with the level of significance, p. GM-SV, gray matter and subcortical lesions- subcortical dilated perivascular spaces; DWM, deep white matter lesions; PV, periventricular white matter lesions; BG, lesions in the basal ganglia and surrounding areas; IT, lesions in the infratentorial regions; GA, global atrophy. [file Image_2.JPEG]

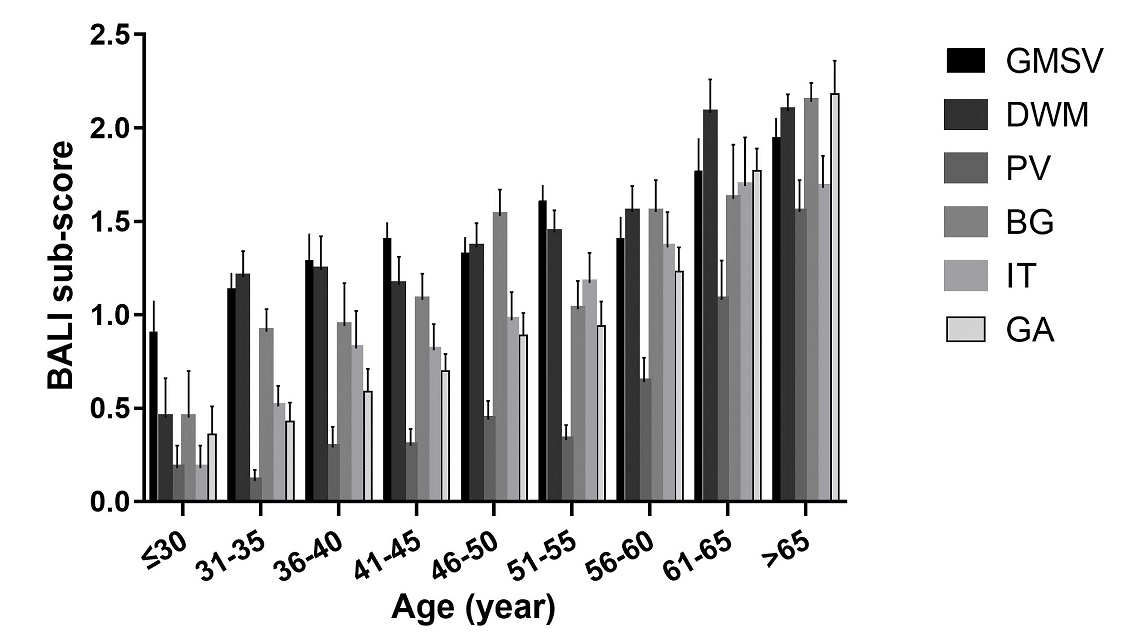

Supplement: FIGURE S3 — The Brain Atrophy and Lesion Index (BALI) subscores by age group. Data are presented as mean ± standard deviation of the subscores of the BALI for each age group. GM-SV, gray matter and subcortical lesions—subcortical dilated perivascular spaces; DWM, deep white matter lesions; PV, periventricular white matter lesions; BG, lesions in the basal ganglia and surrounding areas; IT, lesions in the infratentorial regions; GA, global atrophy. [file Image_3.JPEG]
