# Supplementary material for: Age-Related Whole-Brain Structural Changes in Relation to Cardiovascular Risks Across the Adult Age Spectrum
Source: Front Aging Neurosci. 2019 Apr 24;11:85. doi: 10.3389/fnagi.2019.00085 (PMC6492052; doi:10.3389/fnagi.2019.00085)
Supplement: TABLE S1 — Figure 4A ROC for All subjects (n = 229). Tertile (1): BALI 0-5; Tertile (2): BALI 6-8; Tertile (3): BALI 9-16. *p < 0.05. [file Presentation_1.pdf]

Supplementary Table S1: Figure 4a ROC for All subjects (n=229). Tertile (1): BALI 0-5; Tertile (2): BALI 6-8; Tertile (3): BALI 9-16. *: p<0.05

**Figure 4a-1: tertile (1) vs. Tertile (2)**		**Figure 4a-2: tertile (1) vs. Tertile (3)**		**Figure 4a-3: tertile (2) vs. Tertile (3)**	
N (Tertile 1)	90	N (Tertile 1)	90	N (Tertile 2)	79
N (Tertile 2)	79	N (Tertile 3)	60	N (Tertile 3)	60
AUC	0.616	AUC	0.832	AUC	0.726
Lower Bound	0.531	Lower Bound	0.767	Lower Bound	0.643
Upper Bound	0.701	Upper Bound	0.897	Upper Bound	0.809
Sensitivity	1.000	Sensitivity	1.000	Sensitivity	1.000
	0.911		1.000		1.000
	0.570		0.900		0.900
	0.291		0.600		0.600
	0.089		0.283		0.283
	0.000		0.033		0.033
			0.000		0.000
1-Specificity	1.000	1-Specificity	1.000	1-Specificity	1.000
	0.833		0.833		0.911
	0.411		0.411		0.570
	0.122		0.122		0.291
	0.022		0.022		0.089
	0.000		0.000		0.000
			0.000		0.000
p	0.009*	*p*	<0.001*	*p*	<0.001*

Supplementary Table S2: Figure 4b ROC for Older Group (n=97). Tertile (1): BALI 2-7; Tertile (2): BALI 8-10; Tertile (3): BALI 11-16. *: $p<0.05$

**Figure 4b-1: tertile (1) vs. Tertile (2)**		**Figure 4b-2: tertile (1) vs. Tertile (3)**		**Figure 4b-3: tertile (2) vs. Tertile (3)**	
N (Tertile 1)	35	N (Tertile 1)	35	N (Tertile 2)	35
N (Tertile 2)	35	N (Tertile 3)	27	N (Tertile 3)	27
AUC	0.673	AUC	0.797	AUC	0.655
Lower Bound	0.548	Lower Bound	0.689	Lower Bound	0.519
Upper Bound	0.798	Upper Bound	0.905	Upper Bound	0.791
Sensitivity	1.000	Sensitivity	1.000	Sensitivity	1.000
	1.000		1.000		0.963
	0.829		0.963		0.630
	0.400		0.630		0.296
	0.143		0.296		0.074
	0.000		0.074		0.000
			0.000		
1-Specificity	1.000	1-Specificity	1.000	1-Specificity	1.000
	0.886		0.886		0.829
	0.543		0.543		0.400
	0.257		0.257		0.143
	0.000		0.000		0.000
	0.000		0.000		0.000
			0.000		
p	0.013*	*p*	<0.001*	*p*	0.038*

Supplementary Table S3: Figure 4c ROC for Younger Group (n=132). Tertile (1): BALI 0-4; Tertile (2): BALI 5-6; Tertile (3): BALI 7-11. *: p<0.05

**Figure 4c-1: tertile (1) vs. Tertile (2)**		**Figure 4c-2: tertile (1) vs. Tertile (3)**		**Figure 4c-3: tertile (2) vs. Tertile (3)**	
N (Tertile 1)	54	N (Tertile 1)	54	N (Tertile 2)	38
N (Tertile 2)	38	N (Tertile 3)	40	N (Tertile 3)	40
AUC	0.653	AUC	0.728	AUC	0.584
Lower Bound	0.539	Lower Bound	0.622	Lower Bound	0.457
Upper Bound	0.767	Upper Bound	0.833	Upper Bound	0.711
Sensitivity	1.000	Sensitivity	1.000	Sensitivity	1.000
	0.921		0.925		0.925
	0.500		0.650		0.650
	0.289		0.400		0.400
	0.105		0.200		0.200
	0.000		0.000		0.000
1-Specificity	1.000	1-Specificity	1.000	1-Specificity	1.000
	0.778		0.778		0.921
	0.315		0.315		0.500
	0.056		0.056		0.289
	0.019		0.019		0.105
	0.000		0.000		0.000
p	0.013*	*p*	<0.001*	*p*	0.123
